# Eye-Tracking as a Tool to Evaluate Functional Ability in Everyday Tasks in Glaucoma

**DOI:** 10.1155/2017/6425913

**Published:** 2017-02-15

**Authors:** Enkelejda Kasneci, Alex A. Black, Joanne M. Wood

**Affiliations:** ^1^Department of Computer Science, University of Tübingen, Sand 14, 72076 Tübingen, Germany; ^2^School of Optometry and Vision Science, Institute of Health and Biomedical Innovation, Queensland University of Technology, Brisbane, QLD, Australia

## Abstract

To date, few studies have investigated the eye movement patterns of individuals with glaucoma while they undertake everyday tasks in real-world settings. While some of these studies have reported possible compensatory gaze patterns in those with glaucoma who demonstrated good task performance despite their visual field loss, little is known about the complex interaction between field loss and visual scanning strategies and the impact on task performance and, consequently, on quality of life. We review existing approaches that have quantified the effect of glaucomatous visual field defects on the ability to undertake everyday activities through the use of eye movement analysis. Furthermore, we discuss current developments in eye-tracking technology and the potential for combining eye-tracking with virtual reality and advanced analytical approaches. Recent technological developments suggest that systems based on eye-tracking have the potential to assist individuals with glaucomatous loss to maintain or even improve their performance on everyday tasks and hence enhance their long-term quality of life. We discuss novel approaches for studying the visual search behavior of individuals with glaucoma that have the potential to assist individuals with glaucoma, through the use of personalized programs that take into consideration the individual characteristics of their remaining visual field and visual search behavior.

## 1. Introduction

Glaucoma is one of the main causes of visual field loss in older populations [1], affecting approximately 60 million people worldwide, with the numbers estimated to increase significantly in the future as the population ages [2, 3]. For this reason, the impact of glaucoma on everyday activities such as reading, walking, shopping, or driving, and quality of life has been the focus of numerous research studies [4–12]. Nevertheless, the relationship between functional measures and patients' visual disability in everyday life is still not well understood and requires further research [13].

Many studies have assessed the impact of glaucomatous vision loss on everyday activities through questionnaires or patient-reported outcome measures [8, 9, 14–19], simulators [20–22], or under laboratory conditions [23–26], and some have incorporated measures of visual search behavior. Results from these studies suggest that visual search behavior plays a key role in the ability of individuals with glaucoma to complete everyday activities. More specifically, several studies have reported that some individuals with glaucoma process visual information differently than controls during everyday tasks. For example, Wiecek et al. [27] reported that patients with glaucomatous visual field loss tend to ignore the region of the computer-based image where their scotoma is located, rather than making more eye movements to compensate for their loss. Conversely, another study demonstrated that when viewing dynamic movies of road traffic scenes, glaucoma patients made more fixations and saccades than controls [23]. In a recent study, Crabb et al. [28] showed that visual scanpaths, derived from a passive watching task, can be used to differentiate between individuals with glaucomatous visual field loss and those with no visual field loss. In less dynamic tasks, glaucomatous visual field loss was associated with restricted eye movements; that is, patients performed fewer saccades than controls and viewed different locations of static naturalistic scenes than controls [25, 29]. However, the most valid approach to assessing the functional impairment of patients with glaucoma in everyday activities is by conducting real-world experiments (i.e., observing the person undertaking a particular activity in a field-based environment). However, since such experiments are expensive, time-consuming, and often difficult to standardize, to date few everyday activities have been investigated. Indeed, most of the work on everyday activities has focused on assessing the driving ability and safety of individuals with glaucoma [5–7, 10, 16, 21, 30–32].

Importantly, while the methodological approaches of these studies have varied, they have reached similar conclusions: (1) task performance varies among individuals, (2) glaucomatous field loss does not always lead to poorer performance, and (3) visual field defects related to glaucoma can be compensated for in some individuals through effective head and eye movement strategies. Furthermore, it has been suggested that the results of different studies may relate specifically to that set of circumstances and not reflect individuals' visual behavior in other everyday activities, given that compensatory gaze patterns are highly specific and intrinsically related to the specific task [33]. Furthermore, there appears to be a wide degree of variability in patients' compensatory strategies that are adopted during activities of daily living.

One approach to evaluate the real-world impact of glaucomatous loss and potential compensatory strategies is through assessment of visual search and scanning during daily activities. Assessment of visual search in this way also enables better understanding of the link between visual function and ability, as well as providing a basis for designing training strategies for improvement of daily functioning, and the development of assessment tools for use in a clinical setting.

Eye movements are important in directing gaze and attention towards important task-relevant areas within the visual scene, in order to guide subsequent actions when completing everyday activities [34]. Gaze position identifies where foveal vision is directed towards, known as overt attention. At the same time, attention can also be directed towards peripheral areas of the visual field without reorientating gaze, known as covert attention [35]; when something important is identified in peripheral vision, overt attention can be shifted via a corresponding eye movement. While eye-tracking analysis provides information specifically regarding overt attention, it is also the key technology that helps us in the understanding of visual search and scanning behaviors during daily activities. Importantly, patients with glaucoma may have impaired covert attention capacity, relative to the extent of their visual field loss. Indeed, the ability to simultaneously extract central and peripheral visual information within a single glance, as measured with attentional or useful field of view tests, has been shown to be reduced among older adults with glaucoma, compared to normally sighted controls [36, 37].

Incorporating eye movement analysis in settings that reflect everyday activities is becoming an increasingly popular approach, given that several studies have reported that the ability of patients with glaucoma to perform these activities of daily living is only weakly associated with the extent of their visual field defects, but may be mediated through the complex interaction between field loss and visual scanning strategies. The study of eye movements in glaucoma, particularly in comparison to participants with normal visual fields, is also becoming more common, with advances in eye-tracking technology and analytical approaches making it a more practical approach, particularly for assessing task performance while individuals complete everyday tasks in natural environments.

In this paper, we review existing methods that quantify the effect of glaucomatous visual field defects on the ability to undertake everyday activities through the use of eye movement analysis. Although there is a large body of work investigating eye movements in those with glaucoma, the focus of this narrative review is on studies that have employed eye-tracking while participants complete everyday tasks such as reading, mobility and walking, and driving. We also discuss studies that explored the gaze patterns of individuals with glaucoma while shopping [38], during a face recognition task [26], and making a sandwich [39]. Published studies in peer-reviewed journals were identified through searches using Google Scholar and searches of MEDLINE, PubMed, and Cochrane databases using the following combinations of keywords and phrases: “glaucoma”, “visual field loss”, “eye-tracking”, “eye movements”, “visual search”, “scanpath”, “everyday tasks”, “driving”, “mobility", “walking”, “stepping”, and “shopping”. Studies of other eye conditions causing visual field loss were also considered, where appropriate, to inform future research directions. Relevant studies from these searches were sourced and reviewed and are discussed as appropriate; only studies that were published in English were included.

## 2. Eye-Tracking Technology

The use of eye-tracking as a tool to assess and analyze visual search strategies under real-world conditions is growing, given improvements in eye-tracking technology which make it increasingly applicable to the study of both simple and complex scenarios. Video-based eye-tracking is available as head-mounted and remote technology. Recent developments in head-mounted, mobile eye-tracking technology (e.g., Dikablis Mobile eye-tracker, Pupil Labs eye-tracker, SMI Glasses, and Tobii Glasses) have enabled the study of visual perception and visual behavior in natural environments. Some of these eye-trackers, such as the Dikablis Mobile system, can be worn with spectacles, thus interfering only minimally with the participant's natural viewing behavior. On the other hand, observation and monitoring of scanning behavior can benefit from the use of non-intrusive systems, where cameras are positioned remotely at some distance from the participant.

**Figure 1:**
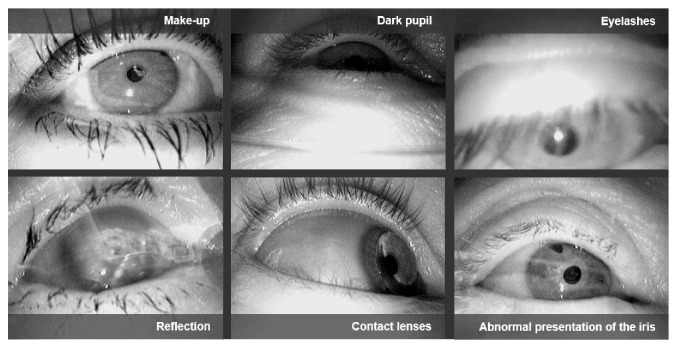
Eye images recorded by mobile, head-mounted eye-trackers in outdoor experiments.

While eye-tracking can be accomplished successfully under laboratory conditions, many studies report difficulties when video-based eye-trackers are employed in natural environments, such as driving [21, 30, 40], shopping [38, 41], or simply walking [42]. The main source of error in such settings is a non-robust pupil signal which primarily arises from challenges in the image-based detection of the pupil. More specifically, a variety of difficulties may occur when using eye-trackers, such as changing illumination (especially problematic when walking outside during the daytime), motion blur, recording errors, and eyelashes covering the pupil (Figure 1). Rapidly changing illumination conditions arise primarily in tasks where the participant is moving rapidly (e.g., while driving), or where the participant rotates relative to unequally distributed light sources. Particularly for older populations, it is important to test the tracking quality of the eye-tracker with the participant's spectacles. Often the tracking rate (i.e., the percentage of video frames where pupil information can be extracted, and consequently, the gaze position can be calculated) and accuracy are significantly degraded when strong illumination and reflections on the spectacle lenses are present. A further issue arises due to the off-axis position of the eye camera in head-mounted eye-trackers. Therefore, studies based on eye-tracking in uncontrolled environments frequently report low pupil detection rates. As a consequence, the data collected in such studies has to be manually post-processed, which is laborious and time-consuming.

Recently, several algorithms have been introduced to tackle these challenges and report very high pupil detection rates in both head-mounted [43–45] and remote eye-tracking [46] technology. Among the state-of-the art algorithms for head-mounted and remote eye-tracking, ExCuSe [43] and ElSe [44], two decision-based approaches based on edge detection and ellipse fitting, show very high accuracy combined with real-time processing capability. When eye-trackers with low sampling rates up to 60 Hz are incorporated, the PupilNet algorithm based on advanced machine learning techniques (i.e., Convolutional Neural Networks), achieves even higher robustness with regard to the above-mentioned sources of noise [47]. The tracking rate is an important parameter and is reported as the proportion of frames where the pupil is detected. It can easily be computed and is usually also reported by the manufacturer's software. The second important parameter is the calibration accuracy, that is, how exactly the position of the participant's gaze is projected into world coordinates (or pixel coordinates in a video for head-mounted devices). Contrary to the tracking rate, a dedicated calibration measurement during the experiment has to be performed, for example, by instructing the participant to fixate on specific markers. As calibration quality is likely to decrease over the duration of the experiment, it is important to assess accuracy before and after the experiment.

Given a reliable eye-tracking signal, several processing steps have to be applied on top of the raw data stream to derive information about visual search behavior. As mentioned in the introductory section, several studies have collected eye movement data on glaucoma patients while they complete everyday tasks, in order to identify their exploratory search patterns. The data recorded in these studies has been mainly analyzed manually and post-experimentally. Basic fixation filters are then applied to extract fixation locations and saccades.

Eye-tracking technology, however, has huge potential beyond that of simply measuring eye movements. Online analysis of eye-tracking data could help to design gaze-based interactive and assistive systems for patients with impaired vision, such as in glaucoma. A crucial prerequisite towards the development of such interactive systems is a robust data analysis pipeline. The first processing step in this pipeline addresses the automated detection of the eye movement type (i.e., fixation, saccade, or smooth pursuit), to extract the spatiotemporal sequence of eye movements (also known as the visual scanpath). Other movements, such as smooth pursuits, microsaccades, ocular drifts, and microtremor, are usually ignored, since it is difficult to extract them from the eye-tracking signal, especially when recorded at low sampling rates (below 120 Hz). For some tasks, information on gaze density in specific areas of interest is sufficient. Such information can be derived from heatmap visualization, as provided by most eye-tracking data analysis software. More sophisticated methods require the examination of a fixation sequence in combination with information from the scene. Several algorithms are available for event detection, such as [54–56], and have been applied in some studies with glaucoma patients. For example, Sippel et al. [38] used advanced data analysis to identify characteristic visual exploration patterns of glaucoma patients during a shopping task. In Kübler et al. [21], such methods were used to investigate eye movement patterns in patients with glaucoma while driving.

To date, most eye movement analytical approaches are based on time-integrated measures, such as the average fixation duration, or the number of fixations directed towards a specific region of interest. Several studies have described such exploratory eye movement patterns in glaucoma patients during everyday tasks. But extracting these at the scanpath level (i.e., the sequence of fixations and saccades) from the large amount of data generated is highly challenging. A manual analysis is very laborious and only applicable to experiments of short duration involving static stimuli (e.g., such as in reading). Dynamic activities such as walking or driving, where the scene is changing with the ego perspective, require automated methods to compare eye-tracking data of different participants (or even more demanding, that of different participant groups), in order to identify common patterns of eye movements, as well as those that differentiate between participant groups. Only a few approaches, such as those based on string similarity [57] which compare scanpaths as a whole, or in segments as described by Kübler et al. [51, 58], can be applied to the analysis of eye-tracking data derived while completing interactive tasks. Such methods are only rudimentarily implemented in most analysis software, yet determining gaze patterns that distinguish between two experimental groups can be highly valuable.

A major issue that needs to be considered prior to undertaking eye-tracking experiments, is the reference coordinate system that the eye-tracker works within. Head-mounted devices record the gaze position relative to the head position (scene video image), which can be challenging to analyze automatically. If the participants move their head, the position of the objects in the video image also changes. Placing easily traceable markers for further image analysis close to relevant objects can speed up data analysis significantly. Remote trackers more commonly provide a gaze vector in a world reference system. Therefore, the exact position of relevant objects with regard to the eye-tracker is helpful to automatically determine whether a certain object was looked at. A relevant issue for recording naturalistic viewing behavior is that the areas over which head movements can be recorded are limited. For tasks that require a large freedom of head movement and rotation, it is possible to combine multiple remote cameras or a head-mounted device and a head tracker. Some eye-trackers also measure head position and orientation within a limited area; for example, the EyeLink tracker can detect a marker placed on the participant's forehead, while Smart Eye fits a head model to multiple camera perspectives.

Recently, eye-tracking has been integrated into virtual reality devices. These have enormous potential to study eye movements in glaucoma, through the provision of ecologically valid measures to individually assess viewing behavior in a well-circumscribed environment.

## 3. Eye Movements and Glaucoma in Everyday Tasks

Table 1 provides a summary of eye-tracking studies that have investigated eye movements of individuals with glaucoma, or other relevant conditions causing visual field loss, while undertaking a range of everyday tasks. The main findings from these studies will be discussed in more detail in the following subsections.

### 3.1. Insights from Reading Experiments

Reading is an everyday task that requires good central vision. Although glaucoma is mainly associated with impaired peripheral vision, many patients also experience paracentral and central visual field loss and difficulties with reading are commonly reported [8, 9, 11, 59, 60]. In support of these self-reported reading difficulties, studies that have measured reading performance in individuals with glaucoma report reduced reading speeds compared to those with normal vision for small size text [61], at low contrast levels [48], or when reading for sustained periods of time [9]. Those individuals with central glaucomatous field loss [62], or who have advanced field loss [9, 63], are also particularly impaired in terms of reading ability. Importantly, as outlined by Crabb [13] in his viewpoint on glaucoma, the reading capacity of those with glaucomatous field loss varies considerably between individuals; studies of eye movements and reading by his research group suggest that differences in eye movement patterns in those with glaucomatous loss may account for some of this variability [48, 49].

Smith et al. [49] reported that reading performance was significantly worse in the eye with more glaucomatous field loss compared to the better eye in a given individual, but that this was not related to the extent of field loss, but rather to measures of contrast sensitivity and visual acuity. Furthermore, those individuals, whose reading speeds were particularly affected in their worse eye, made a larger proportion of backward saccades and “unknown” eye movements (not adhering to expected reading patterns) when reading with this eye in comparison to the better eye [49]. A study by the same research group [50] demonstrated that some of the variability in reading speed in those with advanced glaucomatous loss could be explained by eye movement patterns. A significant association was found between increased saccadic frequency in those with higher reading speeds (for short passages of text) in individuals with glaucoma, which suggested the adoption of compensatory mechanisms to improve task performance. In addition, those who read more slowly tended to read every word in a line (termed text saturation) compared to those with higher reading speeds and controls; these effects were exacerbated during longer periods of sustained reading.

In summary, the incorporation of eye-tracking provides a useful experimental approach for exploring differences in reading performance in those with glaucoma and better understanding of the mechanisms underlying these reading difficulties.

### 3.2. Glaucoma, Mobility, and Walking

Peripheral vision is important for spatial orientation, balance control, and efficient navigation when walking, particularly guiding obstacle avoidance, locomotion planning, and foot placement. Adults with glaucomatous visual field loss have been shown to demonstrate altered balance control when standing [64, 65], along with impaired mobility performance when walking, including slower walking speeds and increased contacts with obstacles, especially in those with bilateral visual field loss [4, 12]. Impaired balance and mobility performance in those with glaucoma is likely to negatively impact on the health and well-being of older adults. For example, greater glaucomatous visual field loss has been linked to reductions in physical activity levels [66], greater levels of fear of falling [67], and increased risk of falls and injuries [5, 68].

Studies have also explored whether specific areas of the visual field are more important for mobility and falls in adults with glaucoma. Murata et al. [69] reported significant associations between central and inferior hemifield regions and self-reported walking difficulties. Other studies also highlight the importance of the inferior visual field region for postural stability [64] and falls risk [68] in glaucoma. These associations are likely to reflect natural human gaze behavior when walking. In uncluttered environments, such as an unobstructed level footpath, gaze is generally directed several steps ahead in the direction of travel to guide route planning and to scan for potential hazards [70, 71]; therefore the inferior visual field area is used to provide important information guiding foot placement and detection of hazards. In more challenging or cluttered environments, where precise foot placement is important for safety, gaze tends to shift towards the stepping locations to optimize stepping accuracy [72].

While inefficient visual scanning of the environment is likely to be an important factor linking visual field loss and impaired mobility and falls in adults with glaucoma, there have been few studies that have assessed the link between eye movements and gaze behavior while walking in individuals with glaucoma. Eye-tracking studies have been undertaken in other ocular conditions with peripheral visual field loss, such as retinitis pigmentosa (RP). Patients with RP have been shown to exhibit narrower horizontal scanning patterns when walking in real environments compared to healthy controls [52], potentially due to the absence of peripheral visual stimulation to trigger eye movements and attention towards these areas. Indeed, recent research using saccadic training has shown promise in improving mobility for RP patients, by consciously directing eye movements and attention outside of the seeing region of the visual field [53]. Further research using robust eye-tracking technology and advanced data analysis, with respect to the dynamic nature of walking, is needed to better understand the eye movement patterns of adults with glaucomatous visual field loss, and explore potential saccadic training paradigms to improve their mobility and quality of life.

### 3.3. Glaucoma and Driving

A large body of work has been conducted over the last two decades to investigate the impact of glaucoma on driving, which has drawn a range of conclusions regarding the impact of glaucoma on driving ability and safety, as summarized in a recent review [73]. Glaucoma has been shown to be an important risk factor for self-reported crashes over the previous 10 years [74–76] and state-recorded crashes [5, 77–80]; however, the underlying reasons for this increased crash risk are unclear. Simulator-based assessments have revealed equivocal results, with some studies reporting increased simulator crashes [81], while others reveal only small differences in performance between those with glaucoma and age-matched controls [20, 21]. On-road performance is also impaired in some drivers with glaucoma compared to those without glaucoma [6, 30–32], with drivers with glaucoma demonstrating difficulties in observation, maintaining their lane position, changing lanes, and planning ahead [31]. Interestingly, while some studies report links between the extent of field loss and driving ability and safety [77, 80, 81], others have failed to find a link [21, 30, 82]. Importantly, few studies have investigated the eye movement patterns of individuals with glaucoma while undertaking driving tasks, which might provide insight into the link between visual field loss and driving ability. Indeed, specific eye movement patterns might act as a compensatory mechanism for the loss of visual function and ultimately provide the basis for effective visual rehabilitation and coping strategies.

In the few on-road studies that have involved eye movements, those glaucoma patients who were rated as safe to drive showed increased exploration activity, in terms of more eccentric head movements, compared to those drivers with glaucoma who were rated as unsafe to drive [21, 30, 83]. Indeed, in a recent study conducted in a driving simulator, driving behavior and gaze patterns of a small group of participants with bilateral glaucoma were investigated by employing recently developed mobile eye- and head-tracking technology [21]. Results from this study demonstrated that those drivers scored as unsafe displayed less eye movements (shorter saccade amplitudes, longer fixation durations, and less fixations), a gaze bias to the right, and a more straight-ahead eye position [21]. The effect of head movements has been shown to be most important in realistic experimental setups and in those driving simulations with a wide field of view which were more representative of the driving scene. Simple driving simulations with a narrow field of view and relatively simple tasks are unlikely to reflect naturalistic viewing behaviors. Differences in eye movement patterns have also been reported in those with glaucoma compared to controls when completing video-based hazard perception tasks [23]. A reduction in saccade rates and smaller number of fixations indicates decreased eye scanning activity, and longer fixation durations appear to be associated with an inability to acquire visual information in a quick and effective manner, as observed in patients who passed the driving assessment in the study by Kübler et al. [21]. Because new information is acquired during fixations, the finding that patients who failed the driving test made fewer saccades suggests that they were unable to process as much of the visual scene as those patients who passed the test. The finding that unsafe glaucoma drivers showed a gaze bias to the right [21] is also in line with Prado Vega et al. [20], who attributed this finding to the optimal control theory of manned-vehicle systems. A possible explanation is that safe glaucoma drivers pay more attention to avoiding traffic hazards (by gaze scanning), whereas unsafe glaucoma drivers attempt to maintain a stable lane position but fail to recognize traffic hazards because of limited gaze compensatory reserves.

### 3.4. Other Everyday Tasks

Very few studies have investigated the link between task performance and eye movements in other everyday tasks.

Glen et al. [26] studied the performance of individuals with advanced glaucoma in a face recognition task and demonstrated that some patients showed good task performance despite their visual field defects. More specifically, the authors found that in patients with bilateral visual defects in the central 10° of their visual field, larger saccades led to better face recognition performance [26]. In contrast, the authors found no significant association between saccade amplitude and task performance in people with normal vision. These findings are in line with several studies described previously, which report that some individuals with glaucomatous visual field loss adopt compensatory eye movements during visual tasks.

Two recent studies, involving the everyday tasks of shopping and sandwich making, provide further interesting insight into this issue. In a real-world shopping task, Sippel et al. [38] compared the functional ability and eye movements of 10 patients with bilateral glaucomatous field loss in comparison to 10 normally sighted subjects. Overall, the glaucoma group took longer to complete the task, yet 8 of the glaucoma patients were able to successfully complete the task within a time frame commensurate with the controls, and showed a significantly higher number of glances towards their visual field defect area. Therefore, systematic exploration of the area of visual field defects seems to be a “time-effective” compensatory mechanism during supermarket shopping, which mirrors the results of on-road driving for those with hemianopic field defects [30, 84].

Recently, Dive et al. [39] showed that while patients with glaucoma were slower than controls to complete naturalistic tasks, such as making a sandwich, as well as an unfamiliar task of building a model, they could still complete these tasks efficiently. Assessment of eye movements while doing these tasks revealed that the glaucoma participants made more head and eye movements and had longer fixation durations compared to the controls; the authors suggested that this may have been a strategy to compensate for reduced visibility when key targets fell within their visual field defects.

## 4. Eye-Tracking as a Means to Assist Individuals with Glaucoma

An interesting research question that arises from the study of eye movements in glaucoma, is whether specific training procedures can assist in the adoption of compensatory gaze patterns in patients with glaucoma that are effective in improving task performance. However, since gaze patterns are task-dependent, it is unclear to what extent eye movement patterns that have been adopted during training on a specific visual search task, can be transferred to real-world tasks, such as driving, walking around, or shopping. For example, Kasneci et al. [30] reported that safe drivers with glaucoma employed a similar viewing strategy in an on-road setting as in a simulated drive [21]. More specifically, the viewing strategy of glaucoma patients who passed the driving tests concentrated on the central 20° visual field area and was combined with frequent but short gazes towards their field defect area and the peripheral visual field. Furthermore, the authors reported that those glaucoma patients who failed the on-road driving test tended to also fail the simulator drive. These researchers investigated task performance and gaze patterns of the same glaucoma group in comparison to normally sighted subjects during a shopping task. Interestingly, there was very high agreement between “good performers” in the driving task and “good performers” in the shopping task, although the compensation strategy employed during shopping differed from that adopted during driving.

In light of these findings, we propose that new methods need to be developed to assess task performance and train and assist glaucoma patients. This is an area where eye-tracking technology could be extremely beneficial. In particular, the combination of eye-tracking and virtual reality offers the potential for evaluating functional ability in glaucoma in complex, yet standardized tasks that mimic everyday tasks. Particularly, in the driving context, this technology could facilitate the systematic assessment of driving safety and viewing behavior during driving. Furthermore, measurements of the visual field could be used to assess individual viewing behavior with respect to the impaired areas in the visual field in an automated way. In this way, personalized training could be developed, for example, by guiding the gaze of an individual towards specific regions through visual or acoustic stimuli.

Moreover, in the driving context, driving assistance systems could utilize unique information regarding an individual driver's eye movements and visual field defects. The design and implementation of such systems is, however, highly challenging, since the visual search behavior (i.e., the visual scanpath) of the driver has to be analyzed in real-time in alignment with objects presented in the dynamically changing driving scene. Kasneci et al. [85] recently introduced a framework based on several machine learning methods to explore hazard perception based on eye movements, where a reliable alignment of gaze and the scene provides the foundation for detection of potentially overlooked traffic hazards. For those cases where the system predicts that the driver has not seen the upcoming hazard, the driver's gaze could be guided towards the hazard by means of visual or acoustic stimuli. If the driver does not react in time, the system should intervene to avoid the collision. Gaze guidance for drivers with visual impairments is particularly challenging, however, as it has to be performed taking into consideration the specific type and location of visual field loss.

In summary, eye-tracking technology is currently a research tool that provides insights into how glaucoma alters attention and viewing behavior. There is huge potential for further development, especially due to advanced analytics that might enable the detection of visual field defects from eye movement recordings during everyday tasks. In recent work, Crabb et al. [28] showed that it might be possible to detect glaucoma during a simple everyday task, such as watching television. Beyond the diagnosis aspects and knowledge of gaze behavior adaptation, it may be possible to design assistive systems that help individuals with glaucomatous visual field loss to maintain or even improve their performance on everyday tasks, increase their independence, and hence improve their long-term quality of life.

## 5. Conclusion

Visual search behavior plays a key role in the ability of individuals with glaucoma to complete everyday activities. With the development of more sophisticated eye-tracking technology, assessment of eye movements is transitioning out of the laboratory to encompass activities such as walking, driving, or other real-world tasks and, hence, provides a powerful tool for better understanding the visual search mechanisms of individuals with glaucoma and their implications for everyday tasks. Combined with virtual reality technology, eye-tracking offers the possibility for focused eye movement research under standardized experimental conditions and the development of personalized solutions to assist glaucoma patients.

## Figures and Tables

**Table 1:** Summary of eye-tracking studies referenced in this work with regard to their participants and eye-tracking devices.

Study	Cohort demographics	Eye-tracker (fps)	Main findings
Burton et al. [48]	53 bilateral glaucoma (mean age 66 ± 9); 40 controls (mean age 69 ± 8)	EyeLink 1000 (1000)	Reduction in reading speed for lower contrast text was greater in glaucoma patients than controls.
Smith et al. [49]	14 bilateral glaucoma (median age 69, IQR 64 to 81)	EyeLink 1000 (1000)	Slower performance and more regression when reading with the worse eye, compared to better eye. Differences in performance not related to magnitude of difference in VF mean deviation index between eyes.
Burton et al. [50]	18 advanced bilateral glaucoma (mean age 71 ± 7); 39 controls (mean age 67 ± 8)	EyeLink 1000 (500)	Similar reading speeds between groups. Some glaucoma patients read slower than controls, partly explained by differences in eye movement behavior.
Prado Vega et al. [20]	23 glaucoma (mean age 65 ± 12); 12 controls (mean age 65.7 ± 9.4)	Smart Eye (60)	Glaucoma patients missed more peripherally projected stimuli during driving in a simulator than controls. Glaucoma patients did not use compensatory visual search patterns.
Kübler et al. [21]	6 binocular glaucoma (mean age 62 ± 7); 8 controls (mean age 602 ± 10)	Dikablis (25)	Glaucoma patients who passed the driving test in the simulator showed increased number of head and gaze movements toward eccentric regions of the VF in comparison to patients who failed.
Crabb et al. [23]	9 binocular glaucoma (mean age 67.6 ± 9.3); 10 controls (mean age 64.4 ± 11.4)	EyeLink (250)	Patients showed different eye movement characteristics (more saccades) than controls when viewing driving scenes in a hazard perception test.
Kasneci et al. [30]	10 binocular glaucoma (mean age 61 ± 9); 10 controls (mean age 60 ± 9)	Dikablis (25)	Patients who passed the on-road driving test focused longer on the central VF and performed more glances towards the area of their VF defect than patients who failed.
Kübler et al. [51]	10 binocular glaucoma (mean age 61 ± 9); 10 controls (mean age 60 ± 9)	Dikablis (25)	Patients can be identified based on their visual scanpath while driving above chance levels.
Sippel et al. [38]	10 binocular glaucoma (mean age 61 ± 9); 10 controls (mean age 60 ± 9)	Dikablis (25)	Patients who showed good performance during supermarket shopping made more glances towards the VF defect area.
Vargas-Martín and Peli [52]	5 retinitis pigmentosa (mean age 58 ± 16); 3 controls (mean age 67 ± 5)	ISCAN (60)	Retinitis pigmentosa patients exhibited narrower scanning strategy than controls.
Ivanov et al. [53]	25 retinitis pigmentosa (mean age 54 ± 13)	Tobii Glasses (30)	An exploratory saccadic training improved search performance, as well as mobility performance.
Dive et al. [39]	12 bilateral glaucoma (mean age 64 ± 15); 13 controls (mean age 73 ± 9)	iViewX^TM^ (50)	Glaucoma patients took longer to complete the task, with longer fixations and more eye and head movements, than controls.
Smith et al. [24]	20 bilateral glaucoma (mean age 67 ± 10); 20 controls (mean age 67 ± 11)	EyeLink II (500)	Glaucoma patients took longer to find targets in photographs.
Crabb et al. [28]	44 glaucoma (median age 69, IQR 63–77); 32 controls (median age 70, IQR 64–75)	EyeLink 1000 (1000)	Differences in signature scanpath patterns when watching television could separate glaucoma from controls.
